# 

**Published:** 1858-04

**Authors:** 


### CONTENTS.

ORIGINAL COMMUNICATIONS— PAGE.

Cases in Practice. By E. Read, M. D .......... 149
Acute Hæmatemesis. By B. F. Schenck, M.D .......... 153
Surgical Cases occurring in the Mercy Hospital of Chicago. Reported by C. N. Ellinwood .......... 157
Case of Erysipelas Neanatorum. By F. K. Bailey, M.D .......... 161
An Unsuccessful Case of Acute Spinal Meningitis. By Dr. Ross C. Russ, 164
Malformation of the Lower Extremities. By D. H. Adam .......... 165

Translations by Dr. Byford—
Sweet Milk a Remedy for Dropsy .......... 167
Collodion as an Erector of Flat, Undeveloped Nipples .......... 170
Valerianate of Ammonia for Epilepsy .......... 170
Glysters of Alum for Dysentery, by Dr. Hamon .......... 170
Belladonna for Incontinence of Urine .......... 171
Expulsion of a Uterine Polypus, by Dr. Bezencenet .......... 171

BOOK AND PAMPHLET NOTICES—

Human Histology. By E. R. Peaslee .......... 172
A Manual of Medical Diagnosis. By A. W. Barclay .......... 172
The Anniversary Discourse before the New York Academy of Medicine. By J. Marion Sims .......... 174
North-American Medico-Chirurgical Review .......... 176
New Journals .......... 177
Editorial Changes .......... 178

EXTRACTS—

Philadelphia County Medical Society .......... 179

EDITORIAL—

American Medical Association and Medical Education .......... 186
Annual Meeting of the Cook Co. Medical Society .......... 190
To Correspondents .......... 190

